# 4 Phenylbutyrate Plus Gene augmentation: A dual therapy To Rescue of SLC6A1 Variant Associated Developmental And Epileptic Encephalopathy

**DOI:** 10.64898/2026.06.05.730491

**Published:** 2026-06-10

**Authors:** Aiden James Delahanty, Kaitlin James, Emma Grace, Ziang Debbie Song, Juexin Wang, Melissa Bassette, Jing-Qiong Kang

## Abstract

**Background/Objectives:**

Pathogenic variants in SLC6A1, which encodes the γ-aminobutyric acid (GABA) transporter GAT-1, cause developmental and epileptic encephalopathies (DEEs) through reduced GABA uptake, impaired transporter trafficking, and functional haploinsufficiency. 4-Phenylbutyrate (PBA) is a clinically available small molecule with chemical-chaperone and histone-deacetylase-inhibitor activities that can rescue misfolded GABAergic proteins, but variant-level rescue data are needed to guide precision treatment.

**Methods:**

We report a newly identified de novo SLC6A1 missense variant, p.Ala305Val (A305V), in a patient with myoclonic-atonic epilepsy and a developmental and epileptic encephalopathy phenotype. A305V was compared with the residue-matched comparator p.Ala305Thr (A305T). Variant effects were evaluated by (i) protein-structure prediction across nine stability-prediction algorithms using the cryo-EM-derived human GAT-1 template (PDB 7Y7W); (ii) 3H-GABA uptake assays in HEK293T cells and in human iPSC-derived astrocytes and cortical neurons; (iii) live-cell confocal microscopy of ER colocalization; (iv) pharmacologic rescue with PBA, TUDCA and salubrinal (v) and GAT-1 cDNA gene-augmentation, alone and in combination with PBA.

**Results:**

AI-based stability predictors uniformly indicated destabilization of GAT-1(A305V) and GAT-1(A305T). A305V reduced ^3^ H-GABA uptake across HEK293T, astrocyte, and neurons. The mutant transporter accumulated within the endoplasmic reticulum (ER), with ER colocalization rising from approximately 30% in wildtype to ∼80% in A305V; PBA reduced ER retention to approximately ∼40% and restored total GAT-1 fluorescence toward wildtype levels. Pharmacochaperones (PBA, TUDCA) restored GABA uptake for the mutant transporters. Wildtype GAT-1 gene augmentation improved mutant GAT-1 uptake and combined PBA-plus-augmentation produced rescue greater than either intervention alone in the available dose-response ranges.

**Conclusions:**

SLC6A1 A305V is a trafficking-impaired, loss-of-function GAT-1 variant whose dysfunction is tractable to two convergent therapeutic axes: pharmacologic correction of folding and trafficking, and augmentation of functional transporter dose. These findings support a two-pronged precision-medicine framework for SLC6A1-related DEEs in which PBA increased the transporter function augmented by increased gene therapy.

**Significance of the study:**

This work links the patient-derived SLC6A1 A305V variant to a defined molecular mechanism—GAT-1 destabilization, ER retention, and reduced GABA uptake—and demonstrates that the deficit is reversible by two independent interventions that converge at the same downstream endpoint of functional surface transporter. Because PBA is already clinically deployable and GAT-1 cDNA augmentation models a future viral or non-viral gene therapy, the combined-rescue logic provides a falsifiable path for precision medicine in SLC6A1-related DEEs: chemical chaperoning corrects the folding bottleneck while transporter augmentation increases the pool available for rescue.

## Full Text Availability

The license terms selected by the author(s) for this preprint version do not permit archiving in PMC. The full text is available from the preprint server.

